# Comparative study of the effect of Thymus daenensis gel 5% and diclofenac in patients with knee osteoarthritis

**DOI:** 10.1051/bmdcn/2019090209

**Published:** 2019-05-24

**Authors:** Morteza Dehghan, Shirin Asgharian, Elena Khalesi, Ali Ahmadi, Zahra Lorigooini

**Affiliations:** 1 Clinical Research Development Unit, kashani Hospital, Shahrekord University of Medical Sciences Shahrekord Iran; 2 Medical Plants Research Center, Basic Health Sciences Institute, Shahrekord University of Medical Sciences Shahrekord Iran; 3 Modeling in Health Research Center, Department of Epidemiology and Biostatistics, School of Public Health, Shahrekord University of Medical Sciences Shahrekord Iran

**Keywords:** Celecoxib, Clinical trial, Diclofenac, Knee osteoarthritis, *Thymus daenensis* gel

## Abstract

Background: Osteoarthritis is a syndrome characterized by joint pain and reduced performance and efficien- cy in patient. *Thymus daenensis* has been used since old times for the treatment of bone and joint deformities and pain in traditional medicine.

Purpose: This study was conducted to examine traditional usages and pharmacological features of *T. daen- ensis* with respect to the effect of the plant in patients with osteoarthritis.

Methods: 120 patients with osteoarthritis were divided into 3 groups. Patients in each group were treated by 5% *Thymus daenensis* gel, 1% diclofenac gel, or placebo for 6 weeks, along with oral celecoxib capsules. Patients were assessed in different intervals, based on the VAS score for assessment of pain in the joint and different dimensions of WOMAC questionnaire.

Results: Pain level (*P* < 0.005), stiffness during the day (*P* < 0.05), morning stiffness (*P* < 0.05) and physi- cal performance (*P* < 0.05) were significantly different among the groups.

Conclusions: *Thymus daenensis* gel improves the symptoms in patients equal and without significant difference than diclofenac group. It can be argued that its use can produce a satisfactory effect on patients with osteoarthritis due to its low cost, easy access, the plant’s natively occurring in Iran.

**Abbreviations**
OA: Osteoarthritis
VAS: Visual Analogue Scale
WOMAC: Western Ontario and McMaster

## Introduction

Osteoarthritis (OA) is a syndrome characterized by joint pain and reduced performance and efficiency in patient [1]. Osteoarthritis is one the most common musculoskeletal disorders. According to several reports, the incidence of knee osteoarthritis has been higher than the incidence of hip osteoarthritis [2–4]. According to the World Health Organization report, 18% of women and 6.9% of men aged over 60 suffer from osteoarthritis [5]. Osteoarthritis treatment is aimed to reduce pain, swelling and disability result- ing from inflammation and an increased quality of life, since there is no decisive cure for it [6]. Some of the solutions used for the treatment of this disease include lifestyle changes, medicinal treatments, physical treatments, and finally surgery interventions [7–9]. In this regard, medicinal treatments including NSAIDs are the main choice for osteoarthritis of the knee [10]. However, adverse side effects of medications [11, 12] as well as the likeli- hood of other diseases cause problems in patients especially in the older patients [13, 14]. Studies have indicated increased tendency of OA patients to use complementary and alternative treatments [15, 16]. These studies show that complementary therapies have wide applications for the treatment of chronic diseases in Iran [17, 18]. One of the complementary therapies used in OA is us- ing herbal medicines. *Thymus daenensis* is one of the effective plants for treatment of osteoarthritis in traditional medicine. *Thy- mus daenensis* fresh leaves and stems have a pleasant smell that results from a combination of compounds in oils and extracts of this plant. In vegetative organs of thyme, a compound such as phenols, tannins, flavonoids, saponins, and bitter substances are found [19–21]. The leaves and flowers of this plant are used as antitussive, antispasmodic and anti-flatulence and to treat colds [22, 23]. *Thymus daenensis* has several properties such as anti- bacterial and antifungal [24–33], and antioxidants [34–42] proper- ties; it also reduces the fat content [43]. It also has anti-inflam- matory properties [44–46] and strengthens the immune system of the body [47], and it inhibits tyrosinase [48]. According to the culture governing the country in higher acceptance of medicinal plants and higher acceptance of them by patients and lower side effects of these drugs, this study was performed to investigate the impacts of topical *Thymus daenensis* gel in treating and control- ling symptoms of osteoarthritis.

## Method

This study has three intervention groups (diclofenac group, herbal gel group and placebo group) with the double-blind clinical trial.

### Ethical considerations

The study design is in compliance with the ethical principles of Helsinki Declaration. This study protocol was approved at the Behavioral Sciences Ethics Committee of Shahrekord Univer- sity of Medical Sciences (ethics code IR.SKUMS.REC.1395.54) and then in the Iranian clinical trial registration center with code IRCT2016112231025N1. Informed consent form was taken from patients participating in the study.

### Plant material and extraction method

*Thymus daenensis* aerial parts were collected from mountain- ous areas of the province in the spring of 2016. Collected Plants were identified by Dr. Shirmardi and a herbarium voucher (no. 248) was deposited at the Herbarium of the Medical Plants Re- search Center of Shahrekord University of Medical Sciences. The powdered plant was extracted by maceration method with Ethanol 70% for 48 h. The obtained extract was passed through filter paper. To remove solvent, the extract was concentrated by rotary evaporator at 37°C and lyophilized by freeze dryer. Then, *Thymus daenensis* gel 5% was formulated in gel base from poly- mer Carbopol-940 as U.S. Pharmacopeia. Diclofenac Gel 1% of Emad-Darman Pars Company and placebo were filled in same aluminum tubes like *Thymus daenensis* gel.

### Standardization of topical gel

The total phenolic content of aqueous *Thymus daenensis* extract was measured using Folin- Ciocalteu. To this end, 1 mg/*ml* crude extract reached a volume of 3 *ml* by addition of distilled water and then combined with 0.5 *ml* Folin-ciocalteu reagent for 10 minutes. Next, 4 *ml* sodium carbonate was added to the resulting mixture. The obtained mixture was stored in the dark for half an hour and the optical absorbance was read at 765nm wavelength. The total phenolic content was measured by using calibration curve, and the results were expressed as the total phenolic content (mg of Gallic acid equivalent/g dried extract) [49]. The total fla- vonoid content Flavonoids were measured according to Liang *et al.* method with some modifications. In short, 5.0 *ml* of each ex- tract solution (0.01 g per 10 *ml* of methanol at 60°C) was mixed with 0.5 *ml* of 2% aluminum chloride and 3 *ml* of 5% potassium acetate. After 40 minutes, the absorption of samples was read against distilled water (blank) at a wavelength 415 nm. While carrying out the experiment, various concentrations of Rutin were prepared. Absorption of samples was compared with a standard curve and total flavonoid content of each extract was stated as mg of Rutin equivalent/g dried extract [50].

### Exclusion criteria

Simultaneous severe diseases such as metabolic and gastrointes- tinal diseases, fluctuation in the dose of the drug during the study, the presence of severe infection, allergy to drugs, pregnancy, lactation, liver abnormal performance tests, history of fracture or surgery on the knee, rheumatoid arthritis and seronegative and se-ropositive arthropathies, intra-articular injection during 6 weeks, simultaneous use of any type of tranquilizer that caused distur- bance in the result were exclusion criteria of study. In addition, patients who had no willingness in the study were excluded from the study.

### Inclusion criteria

Men and women with knee osteoarthritis, diagnosed by an ortho- pedician according to diagnostic criteria, aged 45-75 years who had referred to the Orthopaedic Clinic of Shahrkord University of Medical Sciences at least 3 months before the start of the study, were included in the study. They signed an informed consent to participate in the study.

### Intervention

The first group received 200 mg capsules celecoxib orally and topical gel placebo, the second group received 200 mg capsules celecoxib orally on a daily basis and topical 5% thyme gel and the third group received 200 mg capsules celecoxib orally and topical diclofenac 1% gel 3 times daily for 6 weeks. Patients were instructed that the gel should cover the entire surface of the knee. Topical gel dose for each 5 square centimeters of skin was knuckle. To ensure lack of any additional intervention, patients were asked to not massage the knee.

### Effectiveness evaluation

Patient evaluation was performed by means of the Visual Ana- logue Scale (VAS) to score knee pain at baseline and on the first and third days and first, third, and sixth weeks following the inter- vention. Besides that, the Western Ontario and McMaster Univer- sities Osteoarthritis Index (WOMAC) that is a general and valid instrument to examine OA was used as another measurement tool. This questionnaire examines three dimensions: pain dimension (scored between 0 representing no pain and 4 representing severe pain), physical performance (scored between 0 representing no problem and 4 representing severe problem) and stiffness (scored between 0 representing without stiffness and 4 representing se- vere stiffness). The scores of the dimensions were summed and the mean value was calculated. This questionnaire was evaluated before treatment and during the first, third days and the first, third and sixth weeks after the intervention [21].

### Safety evaluation

All patients were asked to report incidence of allergies or adverse between the three groups. *Chi-*square was used to compare quali- tative variables. Bonferroni test was used for multiple comparisons.

## Results

### Standardization of *Thymus daenensis* gel

The total phenolic content of 1 g of *Thymus daenensis* gel was obtained 5.56 ± 0.98 mg Gallic acid equivalent. Total flavonoid content of 1 g of prepared gel was obtained 0.98 ± 0.06 mg Rutin equivalent. reactions in follow-up intervals. A checklist including several questions about different organs of the body (such as gastrointes- tinal problems, nervous system, respiratory system and skin) was used. Potential adverse reactions were investigated by asking the participants open-ended questions.

### Sampling, randomization and blinding

Non-probability convenient sampling was used to select samples. To this end, the participants were selected from the patients ad- mitted to the clinic. To enroll minimum number of people by tak- ing dropouts into account, 120 people were enrolled in this study who were assigned to three groups of 40 each. Group 1 received placebo gel, group 2 received *Thymus daenensis* extract gel, and group 3 received gel containing diclofenac. To collect data, using convenient sampling method, the 120 participants were assigned to tube A, tube B, and tube C (each containing 40) and by using random sampling method; the patient was asked to select one of the Tube A, B, and C. Then, the intervention was performed. Researchers, patients and statistical analysts were blind to the content of three types of tubes.

### Statistical Analysis

The sample size was obtained 35 people considering 95% confi- dence interval, and test power of 90 percent and study power of 85 percent. Considering drop out, 120 were included in the study. Data were analyzed using STATA software. For data analysis, inferential and descriptive statistics and duplicate data analysis were applied. Difference in pretest and post-test scores between the control and intervention groups were compared. Pain level, morning stiffness of knee joint, improvement in physical perfor- mance and improvement rate stiffness of knee in studied groups were examined. Repeated measurement and One-way analysis of variance (ANOVA) was used to compare quantitative variables

### The baseline characteristics of the study

**Fig. 1:**
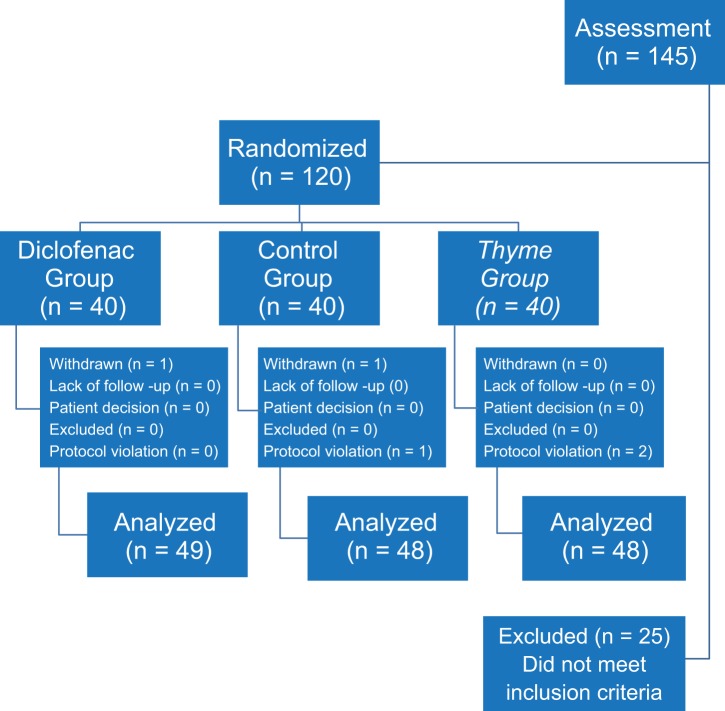
Flow Diagrams of the Randomized Controlled.

The process of registration of patients was conducted from June to August 2016. A total of 120 registered patients were divided into three groups placebo, diclofenac gel, and herbal gel in a ran- dom manner as presented Flow Diagrams (Fig. 1). The average age of the patients was 62.75 ± 8.67 in the range of 45-75 years, and it was 62.6 ± 9.04, 63.83 ± 9.12, and 61.82 ± 7.92 in the pla- cebo, herbal gel, and diclofenac groups, respectively. Out of all patients studied, 51 (43%) were male and 69 people (57%) were female. Regarding age and gender, no significant difference was observed between the groups (*p* = 0.587).

### Clinical responses

**Fig. 2:**
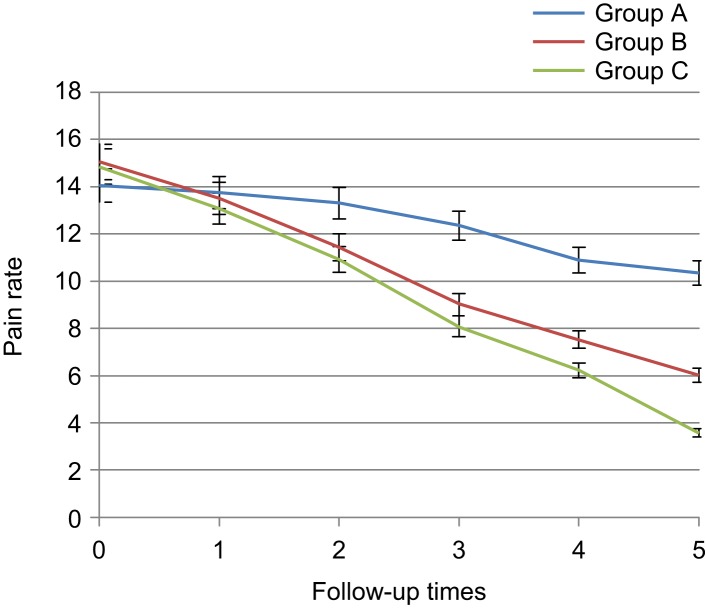
Comparison of knee osteoarthritis patients with respect to knee pain in the 3 groups and during 6 follow- up intervals. A: Placebo, B: *Thymus daenensis* group, C: Diclofenac group.

**Fig. 3:**
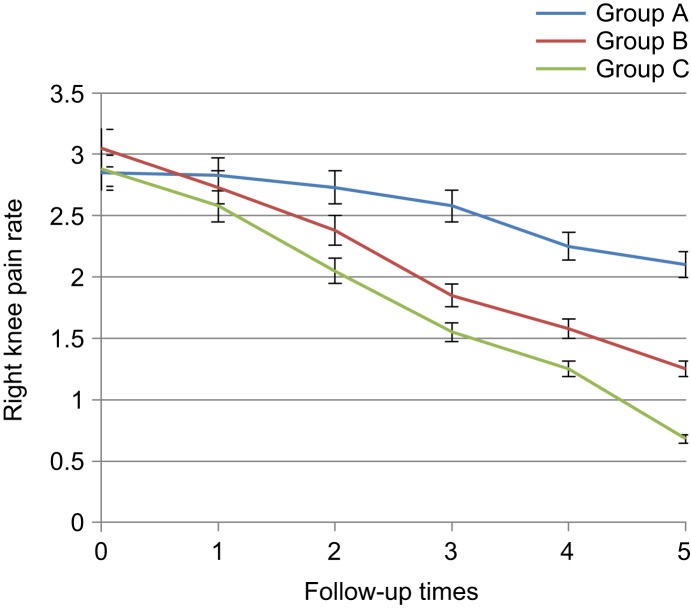
Comparison of knee osteoarthritis patients with respect to right knee pain in the 3 groups and during 6 follow- up intervals. A: Placebo, B: *Thymus daenensis* group, C: Diclofenac group.

Pain at six intervals had overall reduction both in herbal gel group and diclofenac group. On the day before the intervention and the first day after the intervention, significant difference was not found among three groups with respect to pain reduction (14.65 ± 0.77 *vs*. 13.44 ± 0.72, *p* = 0.628) and pain reduction was not sig- nificant, but since the third day, knee pain rate was significantly different between diclofenac and placebo, and between thyme and placebo since the first week (8.05 ± 0.68 *vs.* 12.35 ± 0.79, 9.02 ± 0.67 *vs.* 12.35 ± 0.79, *p* < 0.001) and pain in the diclofenac and thyme groups reduced since that time compared to the placebo group. no significant difference was observed between diclofenac and thyme groups with respect to reduced pain up to the third week after the intervention (6.22 ± 0.62 *vs.* 7.52 ± 0.73, *p* = 0.796), but in the sixth week, a significant difference was observed be- tween two groups (3.57 ± 0.41 *vs.* 6.02 ± 3.87, *p* = 0.02) and rate of reduction in pain was more in diclofenac group (Table 1, Fig. 2). The pain in the right knee since the third day had a significant difference between diclofenac and placebo groups and between placebo and thyme groups since the first week and the reduced pain was observed. In addition, up to the third week, not signifi- cant difference was observed between diclofenac and *Thymus daenensis* groups (1.25 ± 0.13 *vs.* 1.58 ± 0.13, *p* = 0.669), but in the sixth week, a significant difference was observed between the two groups (0.67 ± 0.09 *vs.* 1.25 ± 0.13, *p* = 0.01) that this reduc- tion was more in the diclofenac group (Table 2, Fig. 3).

**Table 1:** Comparison of knee osteoarthritis patients with respect to knee pain in the 3 groups and during 6 follow-up intervals.

	Mean ± SE				*P* value
	Total	A	B	C	
Before starting treatment	14.65 ± 0.77	14.05 ± 0.75	15.05 ± 0.77	14.85 ± 0.80	0.628
First day after treatment	13.44 ± 0.72	13.7 ± 0.73	13.5 ± 0.74	13.07 ± 0.69	0.8
Third day after treatment	11.88 ± 0.74	13.3 ± 0.77	11.42 ± 0.72	10.92 ± 0.68	0.05*
First week after treatment	9.8 ± 0.77	12.35 ± 0.79	9.02 ± 0.67	8.05 ± 0.68	0.000*
Third week after treatment	8.21 ± 0.77	10.9 ± 0.84	7.52 ± 0.73	6.22 ± 0.62	0.000*
Sixth week after treatment	6.65 ± 0.77	10.35 ± 0.82	6.02 ± 0.61	3.57 ± 0.41	0.000*

A: Placebo, B: *Thymus daenensis* group, C: Diclofenac group *P < 0.05.

**Table 2:** Comparison of knee osteoarthritis patients with respect to right knee pain in the 3 groups and during 6 follow-up intervals.

	Mean ± SE			*P* value
	A	B	C	
Before starting treatment	2.85 ± 0.15	3.05 ± 0.17	2.87 ± 0.18	0.668
First day after treatment	2.83 ± 0.15	2.72 ± 0.16	2.57 ± 0.16	0.528
Third day after treatment	2.73 ± 0.16	2.38 ± 0.15	2.05 ± 0.17	0.013*
First week after treatment	2.57 ± 0.17	1.85 ± 0.14	1.55 ± 0.15	0.000*
Third week after treatment	2.25 ± 0.19	1.58 ± 0.13	1.25 ± 0.13	0.000*
Sixth week after treatment	2.1 ± 0.18	1.25 ± 0.13	0.67 ± 0.09	0.000*

A: Placebo, B: *Thymus daenensis* group, C: Diclofenac group *P < 0.05.

**Fig. 4:**
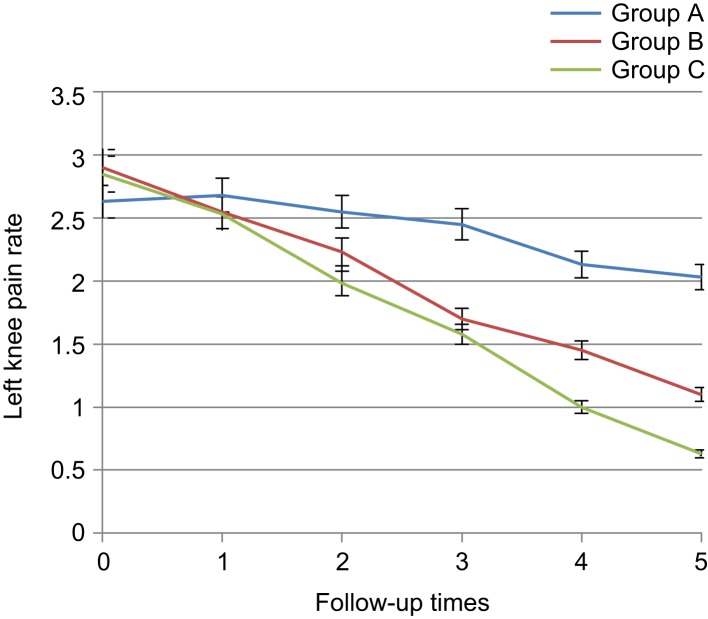
Comparison of knee osteoarthritis patients with respect to left knee pain in the 3 groups during 6 intervals of follow-up. A: placebo group, B: *Thymus daenensis* group, C: diclofenac group.

**Fig. 5:**
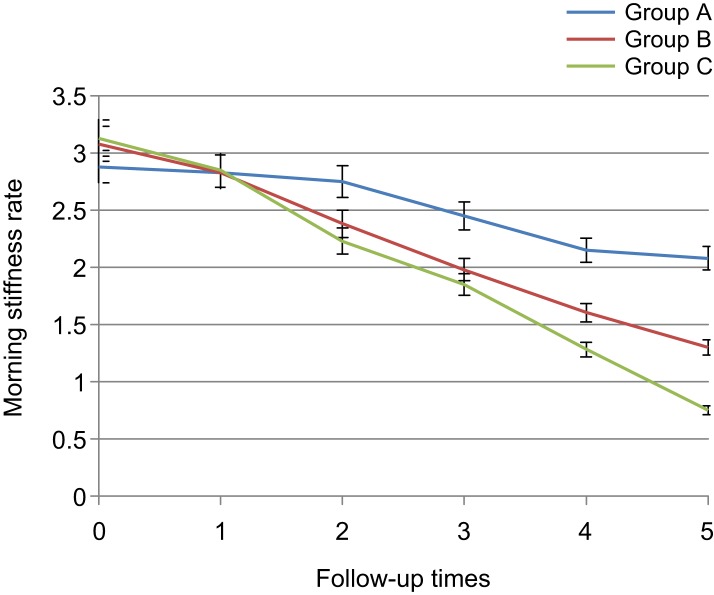
Comparison of knee osteoarthritis patients with respect to morning stiffness in the 3 groups during 6 intervals of follow-up. A: Placebo group, B: *Thymus daenensis* group, C: Diclofenac group.

The pain in the left knee since the third day had a significant difference between diclofenac and placebo groups and between placebo and thyme groups since the first week and the reduced pain was observed. Besides that, up to the third week, no sig- nificant difference was observed between diclofenac and *Thymus daenensis* groups (1 ± 0.13 *vs.* 1.45 ± 0.12, *p* = 0.587), but in the sixth week, a significant difference was noted between the two groups (± 0.09 *vs.* 1.1 ± 0.12, *p* = 0.04) that this reduction was more in the diclofenac group (Table 3, Fig. 4). Morning stiffness rate declined both in herbal gel and diclofenac groups. The morning stiffness had no significant difference among the three groups before the intervention and the first day after the intervention (3.02 ± 0.14 *vs.* 2.83 ± 0.14, *p* = 0.903), but with reduction in morning stiffness, since the third day, significant difference was observed between diclofenac and placebo groups and between thyme and placebo groups since the first week (1.85 ± 0.15 *vs.* 2.45 ± 0.18, 1.97 ± 0.13 *vs.* 2.45 ± 0.18, *p* < 0.01). In addition, no significant difference was observed between diclofenac and *Thymus daenen- sis* groups up to the third week after intervention (1.28 ± 0.0.13 *vs.* 1.6 ± 0.13, *p* = 0.822), but in the sixth week, the two groups also had significant differences (0.75 ± 0.09 *vs.* 1.3 ± 0.13, *p* = 0.01) and the reduction of morning stiffness in the diclofenac group was higher (Table 4, Fig. 5).

**Table 3:** Comparison of knee osteoarthritis patients with respect to left knee pain in the 3 groups during 6 intervals of follow-up.

	Mean ± SE			*P* value
	A	B	C	
Before starting treatment	2.62 ± 0.17	2.9 ± 0.16	2.85 ± 0.15	0.424
First day after treatment	2.68 ± 0.17	2.55 ± 0.15	2.53 ± 0.12	0.754
Third day after treatment	2.55 ± 0.17	2.23 ± 0.13	1.97 ± 0.15	0.025*
First week after treatment	2.45 ± 0.17	1.7 ± 0.13	1.58 ± 0.12	0.000*
Third week after treatment	2.13 ± 0.18	1.45 ± 0.12	1 ± 0.13	0.000*
Sixth week after treatment	2.03 ± 0.18	1.1 ± 0.12	± 0.09	0.000*

A: Placebo, B: *Thymus daenensis* group, C: Diclofenac group *P < 0.05.

**Table 4:** Comparison of knee osteoarthritis patients with respect to morning stiffness in the 3 groups during 6 intervals of follow-up.

	Mean ± SE			*P* value
	A	B	C	
Before starting treatment	2.88 ± 0.15	3.07 ± 0.13	3.12 ± 0.16	0.449
First day after treatment	2.83 ± 0.14	2.83 ± 0.14	2.85 ± 0.15	0.99
Third day after treatment	2.75 ± 0.16	2.38 ± 0.13	2.22 ± 0.14	0.013*
First week after treatment	2.45 ± 0.18	1.97 ± 0.13	1.85 ± 0.15	0.019*
Third week after treatment	2.15 ± 0.18	1.6 ± 0.13	1.28 ± 0.13	0.000*
Sixth week after treatment	2.07 ± 0.17	1.3 ± 0.13	0.75 ± 0.09	0.000*

A: Placebo, B: *Thymus daenensis* group, C: Diclofenac group *P < 0.05.

**Fig. 6:**
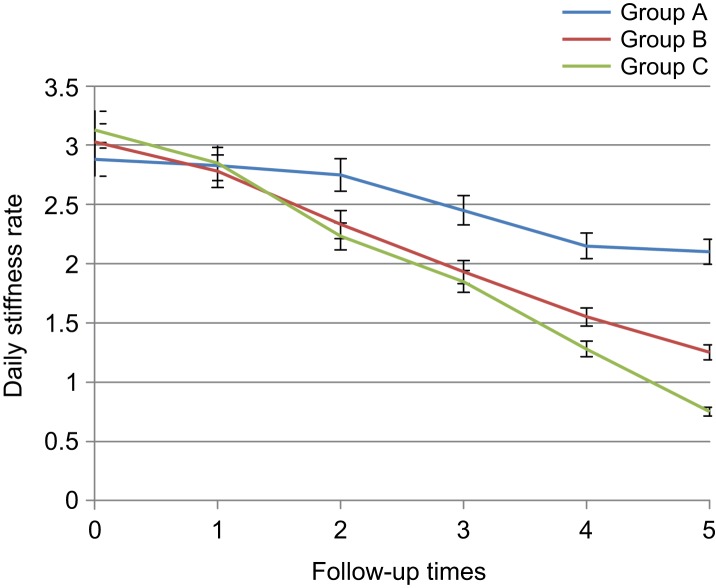
Comparison of knee osteoarthritis patients with respect to daily stiffness rate in the 3 groups during 6 intervals of follow-up. A: Placebo group, B: *Thymus daenensis* group, C: Diclofenac group.

**Fig. 7:**
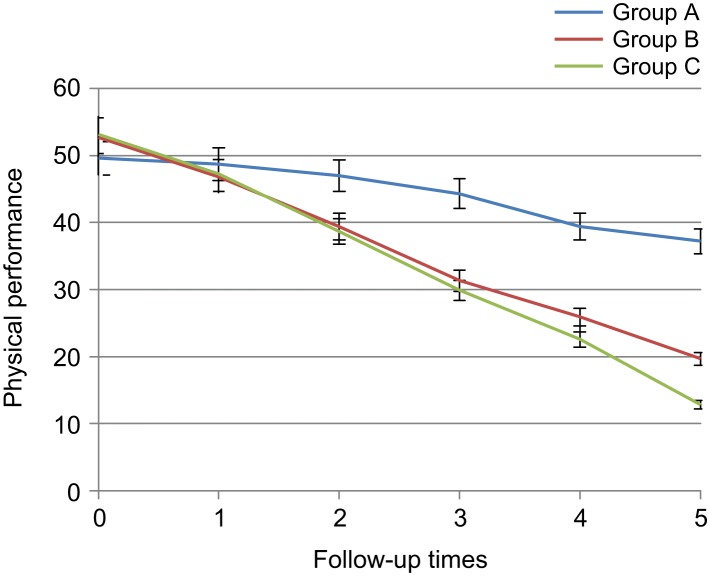
Comparison of knee osteoarthritis patients with respect to physical performance in the 3 groups during 6 intervals of follow-up. A: placebo group, B: *Thymus daenensis* group, C: diclofenac group.

Regarding daily stiffness rate, significant difference was not found among three groups before intervention and first day after intervention (3.01 ± 0.15 *vs.* 2.81 ± 0.14, *p* > 0.05), but since the third day, significant difference was noted between diclofenac and placebo groups(2.22 ± 0.14 *vs.* 2.75 ± 0.16, *p* < 0.01), and since the first week, significant difference was observed between two thyme and placebo groups (1.93 ± 0.13 *vs.* 2.45 ± 0.18, *p* < 0.01). In these groups, pain reduction rate in diclofenac and herbal gel groups was more than the placebo group. In addition, no signifi- cant difference was observed between diclofenac and *Thymus daenensis* groups up to the third week after intervention (1.28 ± 0.13 *vs.* 1.55 ± 0.13, *p* > 0.05). However, daily stiffness reduction in the diclofenac group was higher in sixth week (0.75 ± 0.09, *p* < 0.02) (Table 5, Fig. 6). Both in the herbal gel group and diclofen- ac group, physical performance improved. Regarding physical performance in one day before the intervention and the first day after the intervention, significant difference was not found among the three groups (51.94 ± 2.63 *vs.* 47.71 ± 15.45, *p* > 0.05), but since the third day, significant difference was noted between diclofenac and placebo groups (38.67 ± 2.36 *vs.* 47 ± 2.56, *p* < 0.05) and between thyme and placebo group since the first week (32.17 ± 2.24 *vs.* 44.02 ± 2.78, *p* < 0.05) and physical perfor-mance improvement was seen in the group receiving diclofenac and the group received herbal gel. No significant difference was observed between two diclofenac and thyme groups with respect to physical performance index up to end of the intervention (12.85 ± 1.46 *vs.* 20.85 ± 2.24, *p* > 0.05) and in both groups, physical performance improvement was equal (Table 6, Fig. 7).

**Table 5:** Comparison of knee osteoarthritis patients with respect to daily stiffness rate in the 3 groups during 6 intervals of follow-up.

	Mean ± SE			*P* value
	A	B	C	
Before starting treatment	2.88 ± 0.15	3.03 ± 0.14	3.12 ± 0.16	0.492
First day after treatment	2.83 ± 0.14	2.77 ± 0.15	2.85 ± 0.15	0.934
Third day after treatment	2.75 ± 0.16	2.32 ± 0.13	2.22 ± 0.14	0.027*
First week after treatment	2.45 ± 0.18	1.93 ± 0.13	2.85 ± 0.15	0.014*
Third week after treatment	2.15 ± 0.18	1.55 ± 0.13	1.28 ± 0.13	0.000*
Sixth week after treatment	2.1 ± 0.17	1.25 ± 0.12	0.75 ± 0.09	0.000*

A: Placebo, B: *Thymus daenensis* group, C: Diclofenac group *P < 0.05.

**Table 6:** Comparison of knee osteoarthritis patients with respect to physical performance in the 3 groups during 6 intervals of follow-up.

	Mean ± SE				*P* value
	Total	A	B	C		
Before starting treatment	51.94 ± 2.63	49.57 ± 2.56	53.47 ± 2.54	53.12 ± 2.80	0.514
First day after treatment	47.71 ± 2.44	48.67 ± 2.47	47.72 ± 2.38	47.22 ± 2.55	0.915
Third day after treatment	41.82 ± 2.47	47.02 ± 2.56	40.27 ± 2.32	38.67 ± 2.36	0.038*
First week after treatment	35.17 ± 2.72	44.02 ± 2.78	32.17 ± 2.24	29.87 ± 2.56	0.000*
Third week after treatment	29.43 ± 2.73	39.42 ± 2.96	26.82 ± 2.24	22.55 ± 2.22	0.000*
Sixth week after treatment	23.33 ± 2.72	37.66 ± 2.92	20.85 ± 2.24	12.58 ± 1.46	0.000*

A: Placebo, B: *Thymus daenensis* group, C: Diclofenac group *P < 0.05.

**Fig. 8:**
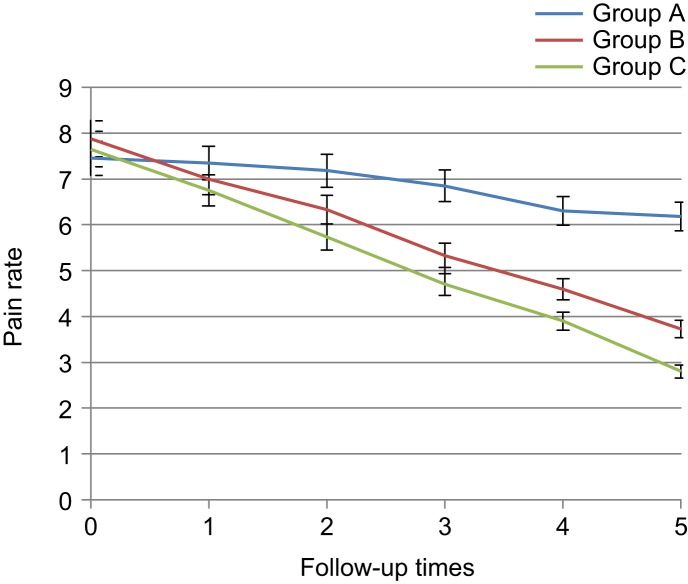
Comparison of knee osteoarthritis patients with respect to pain rate in the 3 groups during 6 intervals of follow-up based on VAS criterion. A: Placebo group, B: *Thymus daenensis* group, C: Diclofenac group.

The knee pain rate based on VAS criterion had no significant difference before intervention and first day after the intervention (7.67 ± 0.33 *vs.* 7.04 ± 0.31, *p* > 0.05), but a significant difference was noted between placebo and diclofenac groups since the third day (7.18 ± 0.33 *vs.* 5.72 ± 0.28, *p* < 0.05) and between placebo and thyme groups since first week (6.85 ± 0.34 *vs.* 5.45 ± 0.31, *p* < 0.05) and a reduction in pain was observed. In addition, two groups of diclofenac and *Thymus daenensis* had no significant different up to a third week (3.9 ± 0.25 *vs.* 4.75 ± 0.34, *p* > 0.05), but since the sixth week, the difference was significant between the groups (2.78 ± 0.25 *vs.* 4.1 ± 0.33, *p* < 0.05) and pain reduc- tion in the diclofenac group was higher (Table 7, Fig. 8).

**Table 7:** Comparison of knee osteoarthritis patients with respect to pain rate in the 3 groups during 6 intervals of follow-up based on VAS criterion.

	Mean ± SE				*P* value
	Total	A	B	C	
Before starting treatment	7.67 ± 0.33	7.45 ± 0.32	7.88 ± 0.34	7.65 ± 0.33	0.502
First day after treatment	7.04 ± 0.31	7.35 ± 0.32	7 ± 0.32	6.75 ± 0.30	0.317
Third day after treatment	6.41 ± 0.33	7.18 ± 0.33	6.38 ± 0.33	5.72 ± 0.28	0.006*
First week after treatment	5.67 ± 0.34	6.85 ± 0.34	5.45 ± 0.31	4.7 ± 0.28	0.000*
Third week after treatment	4.98 ± 0.35	6.3 ± 0.33	4.75 ± 0.34	3.9 ± 0.25	0.000*
Sixth week after treatment	4.35 ± 0.38	6.18 ±v0.35	4.1 ± 0.33	2.78 ± 0.25	0.000*

A: Placebo, B: *Thymus daenensis* group, C: Diclofenac group *P < 0.05.

Comparison Means and 95% Confidence Intervals (CIs) of main outcomes in osteoarthritis patients before and after treatment was presented in Table 8.

**Table 8:** Comparison Means and 95% Confidence Intervals (CIs) of outcomes in osteoarthritis patients before and after treatment.

Outcome	Groups	Initial		Final	
		Mean ± SD	CI 95%	Mean ± SD	CI 95%
Knee Pain	A	14.05 ± 4.75	12.53-15.56	10.35 ± 5.17	8.69-12.00
	B	15.05 ± 4.85	13.49-16.6	6.02 ± 3.87	4.78-7.25
	C	14.85 ± 5.06	013.23-16.46	3.57 ± 2.6	52.73-4.40
Right Knee Pain	A	2.85 ± 0.97	2.53-3.16	2.1 ± 1.12	1.74-2.45
	B	3.05 ± 1.1	2.69-3.4	1.25 ± 0.84	0.98-1.5
	C	2.87 ± 1.15	02.50-3.23	0.67 ± 0.57	10.48-.085
Left Knee Pain	A	2.62 ± 1.05	2.28-2.95	2.03 ± 1.12	1.67-2.38
	B	2.9 ± 0.98	2.58-3.2	1.1 ± 0.77	0.85-1.3
	C	2.85 ± 0.94	12.54-3.15	0.59 ± 0.58	40.4-0.77
Morning Stiffness	A	2.88 ± 0.93	2.58-3.17	2.07 ± 1.09	1.72-2.41
	B	3.07 ± 0.85	2.79-3.3	1.3 ± 0.82	1.03-1.5
	C	3.12 ± 0.99	42.80-3.43	0.75 ± 0.54	60.57-0.92
Daily Stiffness	A	2.88 ± 0.93	2.58-3.17	2.1 ± 1.1	1.74-2.45
	B	3.03 ± 0.89	2.74-3.3	1.25 ± 0.77	1.00-1.4
	C	3.12 ± 0.99	12.80-3.43	0.75 ± 0.54	90.57-0.92
Physical Performance	A	49.57 ± 16.21	44.38-54.75	37.66 ± 18.67	31.68-44.63
	B	53.47 ± 16.08	48.32-58.6	20.85 ± 14.41	16.24-25.4
	C	53.12 ± 17.72	147.45-58.71	2.85 ± 9.2	59.9-15.7
Pain Rate Based on VAS Criterion	A	7.33 ± 2.08	6.66-7.99	6.05 ± 2.18	5.35-6.74
	B	7.78 ± 2.16	7.08-8.4	3.68 ± 2.3	2.94-4.4
	C	7.65 ± 2.05	76.99-8.30	2.18 ± 1.08	11.83-2.52

### Short term tolerance and safety

In the diclofenac group, no topical or systematic adverse effects were reported. In addition, thyme gel was accepted well by pa- tients and no side effects or adverse physical effect was seen in follow- ups.

## Discussion

This study examines the effect of *Thymus daenensis* extract gel on knee pain and performance of patients with osteoarthritis. Our study showed that knee pain of patients in the group consuming the *Thymus daenensis* gel significantly decreased so that knee pain reduction in thyme group was equal and without significant difference than diclofenac group up to fifth interval or third week after intervention. This result may be due to the characteristics of the compounds in the extract of this plant. For example, there is flavonoid compound in this extract and various studies such as the study conducted by Asgari *et al.* have shown that these com- pounds can reduce the production of arachidonic acid through Inhibition of cyclooxygenase and lipoxygenase [46]. Substances resulting from the oxidation of arachidonic acid cause pain and inflammation in the tissue [51], which in turn will intensify the pain. As a result, flavonoids reduce the pain and inflammation. Research has also proven that these compounds can act as ligands for opioid receptors and directly exert their analgesic effect [52]. Since the effects of oxidative stress and the products of oxidative reactions on the creation of osteoarthritis have been proven, anti- oxidant effects of this plant can be possible causes of the observed effects, especially that effects of topical antioxidants containing vitamin C and E, selenium, zinc, isoflavones and polyphenols in tea in inhibiting oxidative stress processes in the skin have been demonstrated [53]. This suggests that topical application of anti- oxidants can also be effective. Free radicals of oxygen produced in the progression of this disease in joint, indirectly through caus- ing tissue damage and direct effect on receptors causing pain, stimulate the neuroendocrine system [54].

The results obtained in the case of morning stiffness and stiff- ness of knee joint of patients during the day also indicated that there is *Thymus daenensis* gel reduces the stiffness of the knee joint that this reduction was equal to the fifth interval of follow- up with diclofenac. In the osteoarthritis disease, created inflam- mation leads to the creation of joint stiffness that our studied plant applies its anti-inflammatory effects through various mechanisms. The substances available in the plant extracts such as phenolic acids, alkaloids, tannins and saponins might be involved in this effect of thyme [20, 21, 55]. For example, in a study conducted by Cichocki *et al.* on the effect of several types of phenolic acids on mouse epidermal cells, it was observed that these compounds inhibited COX-2 activation. One of the most important enzymes that is involved in the production of inflammatory mediators such as prostaglandins, prostacyclin and thromboxane is cyclooxyge- nase whose inhibition inhibits the inflammatory pathways and loss of pain and inflammatory symptoms [56]. Another study by Singh *et al.* on cancerous cells confirmed the effect of isoquino- line alkaloid on the decreased expression of cyclooxygenase-2 and reduced production of prostaglandin E2 and prostaglandin E2 receptors [57]. In the case of physical performance of patients, physical activity significantly improved in both groups received diclofenac and *Thymus daenensis* that significant difference was not found between two groups to end of the study. This can be due to a reduction in knee pain of patients during daily activities and improvement in other symptoms of the disease by reducing inflammation.

### Limitations of study

Although the effectiveness of the thyme gel was approved in this study, there are some limitations to generalize these results to the entire population, including the small size of the population used in this study. Lack of objective criteria for estimating the perfor- mance of the patients, despite validity and reliability of WOMAC questionnaire was another important limitation of the study. In addition, since the osteoarthritis is a disease with chronic nature, the study of variables at an interval after the intervention could be a limitation of our study. With increasing the period of study, we can have a better estimate of the impact and acceptability and safety of the gel. In addition, it determines side effects and topi- cal and general effects resulting from continuous using of the considered drug well.

## Conclusion

Based on the results of this study, the medicinal plant thyme could effectively reduce knee pain, morning stiffness and daily stiffness in the knees and improve physical performance of patients. In our study, no side effect for this plant was reported, so it can be ar- gued that its use can produce a satisfactory effect on patients with osteoarthritis due to its low cost, easy access, the plant’s natively occurring in Iran and no problem caused by the consumption of chemical drugs. It is suggested that be done further studies with different doses of the plant to be conducted to find the dose that has the highest impact and lowest side effect.
